# Perinatal loss in Tanzania: Perspectives of maternal-child healthcare providers

**DOI:** 10.1371/journal.pgph.0003227

**Published:** 2024-05-20

**Authors:** Sharla Rent, Raziya Gaffur, Getrude Nkini, Enna Geofrey Sengoka, Pendo Mlay, Cheryl A. Moyer, Monica Lemmon, Sharron L. Docherty, Blandina T. Mmbaga, Catherine A. Staton, Aisa Shayo

**Affiliations:** 1 Department of Pediatrics, Duke University School of Medicine, Durham, North Carolina, United States of America; 2 Duke Global Health Institute, Durham, North Carolina, United States of America; 3 Department of Obstetrics and Gynecology, Kilimanjaro Christian Medical Center, Moshi, Tanzania; 4 Kilimanjaro Christian Medical University College, Moshi, Tanzania; 5 Department of Obstetrics and Gynecology, University of Michigan, Ann Arbor, Michigan, United States of America; 6 Department of Learning Health Sciences, University of Michigan, Ann Arbor, Michigan, United States of America; 7 Department of Population Health Sciences, Duke University, Durham, North Carolina, United States of America; 8 School of Nursing, Duke University, Durham, North Carolina, United States of America; 9 Kilimanjaro Clinical Research Institute, Moshi, Tanzania; 10 Department of Emergency Medicine, Duke University School of Medicine, Durham, North Carolina, United States of America; PLOS: Public Library of Science, UNITED STATES

## Abstract

Over 98% of stillbirths and neonatal deaths occur in Low- and Middle-Income Countries, such as Tanzania. Despite the profound burden of perinatal loss in these regions, access to facility or community-based palliative and psychosocial care is poor and understudied. In this study we explore perinatal loss through the lens of front-line healthcare providers, to better understand the knowledge and beliefs that guide their engagement with bereaved families. A Knowledge Attitudes and Practices survey addressing perinatal loss in Tanzania was developed, translated into Swahili, and administered over a 4-month period to healthcare professionals working at the Kilimanjaro Christian Medical Center (KCMC). Results were entered into REDCap and analyzed in R Studio. 74 providers completed the survey. Pediatric providers saw a yearly average of 5 stillbirths and 32.7 neonatal deaths. Obstetric providers saw an average of 11.5 stillbirths and 13.12 neonatal deaths. Most providers would provide resuscitation beginning at 28 weeks gestational age. Respondents estimated that a 50% chance of survival for a newborn occurred at 28 weeks both nationally and at KCMC. Most providers felt that stillbirth and neonatal mortality were not the mother’s fault (78.4% and 81.1%). However, nearly half (44.6%) felt that stillbirth reflects negatively on the woman and 62.2% agreed that women are at higher risk of abuse or abandonment after stillbirth. A majority perceived that women wanted hold their child after stillbirth (63.0%) or neonatal death (70.3%). Overall, this study found that providers at KCMC perceived that women are at greater risk of psychosocial or physical harm following perinatal loss. How women can best be supported by both the health system and their community remains unclear. More research on perinatal loss and bereavement in LMICs is needed to inform patient-level and health-systems interventions addressing care gaps unique to resource-limited or non-western settings.

## Introduction

Worldwide, there are approximately 2.4 million neonatal deaths [1] and 2.6 million stillbirths each year [2]. More than 98% of all perinatal loss occurs in low- and middle-income countries (LMICs) [2], with over 75% of neonatal deaths occurring in sub-Saharan Africa (SSA) [3]. Perinatal loss is a unique health marker, consisting of both neonatal mortality and stillbirth. Neonatal mortality encompasses the death of a liveborn infant before 28 days of life while stillbirth represents the loss of a fetus before delivery. While variation exists in definitions of “perinatal loss”, professional pediatric organizations suggest that the term should include infant deaths that occur at less than 28 days of age and fetal deaths at a gestational age of 20 weeks or more [4]. In some settings, including global data tracking of neonatal mortality and stillbirth by the World Health Organization (WHO), this definition is restricted to gestational ages 28 weeks or higher [5]. In this paper, we use “perinatal loss” to refer to stillbirth at or after 28 weeks gestation or death of a liveborn infant within the first 28 days after delivery. Globally, the burden of perinatal loss persists despite overall improvement in mortality for young children, with neonatal mortality now comprising almost half of all global deaths under age five [6]. Tanzania carries a disproportionate burden of this perinatal loss; Tanzania is among the top 10 countries for neonatal death [1] and ranks 9th globally in total annual stillbirths [2]. Furthermore, nearly half of mothers in Tanzania experience the loss of at least one child by the end of their childbearing years [7].

Neonatal mortality has been a key global health issue for decades, featured in both the Millenium Development Goals [8] and the subsequent Sustainable Development Goals [9]. Recently, stillbirth has increasingly been recognized as a key metric of population health [5,10]. Parallel to this global recognition has been increased efforts to classify [11] and report stillbirth deliveries [12,13]. Arriving at an international definition of “stillbirth” has been challenging, with over 80 classification systems reported in the literature in the past two decades [14]. This plethora of classification systems has hampered efforts to track and compare stillbirth rates and causes between settings. The influence of gestational age on perinatal loss classification has also posed a challenge for researchers and clinicians. Notably, the WHO’s threshold of 28-weeks’ gestation for international reporting of stillbirth and neonatal mortality [15] has falsely been extrapolated as representing a practice guideline offered by the WHO [16]. This extension of a research threshold into clinical practice can impact which infants receive resuscitation and how deaths are classified. Historically, underreporting, or misreporting, perinatal loss has impeded appropriate characterization of the global burden and, thus, the ability for clinicians and researchers to target interventions to bereaved families [17].

While African nations have the highest stillbirth and neonatal mortality rates, scant literature exists on support for affected families [18]. Published studies highlight how women who experience loss during childbearing are more vulnerable to social and physical harm [19]. These risks are exacerbated in settings with high rates of stigma associated with perinatal loss [20], social prioritization of fertility [21], and poor social support mechanisms following loss. Despite the profound burden of perinatal loss on families and communities in these regions, access to facility or community based palliative and psychosocial care is poor and understudied [22,23]. Indeed, the bulk of existing work on supporting families following perinatal loss is primarily derived from an Anglo-centric cultural context [24–27], in stark contrast to the communities in which most neonatal loss and stillbirth occur. Given the widespread exposure to perinatal loss in Tanzania, and other LMICs, how local healthcare providers conceptualize and approach this important subset of child loss is an important area of investigation. By studying perinatal loss through the lens of front-line healthcare providers, we can better understand the foundation from which they engage with families. Knowledge gained in this area can then inform interventions that have direct benefit to families.

The high concentration of perinatal loss, significant resource constraints, and underutilization of bereavement tools in LMICs provide a rich research environment in which to examine, and address, unmet support needs surrounding perinatal loss. In this study, we explore provider beliefs and perceptions about perinatal loss classification, gestational age thresholds for intervention, expected survival rates based on gestational age, stigma, and parental wishes surrounding loss. Through the use of tailored Knowledge Attitudes and Practices (KAP) survey at a high-volume tertiary care referral center in the Kilimanjaro region of Tanzania we hope to better understand the perspectives of hospital-based maternal and child healthcare providers regarding perinatal loss and its consequences for women.

## Methods

We used the KAP survey to explore provider exposure to stillbirth and neonatal death, facility-level definitions of stillbirth, expectations for survival based on gestational age, as well as perceptions and beliefs about perinatal loss.

### Study context

Survey administration for this study took place at the Kilimanjaro Christian Medical Center (KCMC), a large referral and teaching hospital in Moshi, Tanzania that serves more than 13 million people from 4 primary regions: Kilimanjaro, Arusha, Tanga, and Manyara [28]. Moshi itself is an urban town with greater than 200,000 residents in Northern Tanzania along the border of Kenya and the Kilimanjaro National Park. Over 250 outlying health facilities refer patients to KCMC so that they can receive a higher level of care. Across the Kilimanjaro region, women commonly get two antenatal ultrasounds–one during the second trimester and one during the third trimester. If there is clinical concern for an in-utero fetal demise, women would get an additional ultrasound to assess for fetal loss. The antenatal care and post-natal follow-up visits for women are conducted by specialists or residents within the department of Obstetrics and Gynecology.

KCMC has an annual delivery volume of approximately 4000 infants per year [29]. At the time of this study, there were 8 pediatricians, 13 obstetricians, and 7 midwives at KCMC. The Pediatric and Obstetrics and Gynecology Departments both run residency programs with training occurring at KCMC. Midwives in the labor ward are responsible for the initial resuscitation of normal deliveries. Specialists and resident physicians from the pediatric department attend higher-risk deliveries. Sick infants are admitted to the Neonatal Intensive Care Unit (NICU), which has 6 high-intensity beds, or one of three lower acuity newborn care units with a total capacity of 100 neonatal beds. Approximately 15% of births at KCMC are transferred to these units for higher-level care [28]. The NICU has one mechanical ventilator; continuous positive airway pressure (CPAP) devices are available for respiratory support. Infants are cup-fed or fed via a nasogastric tube if they are unable to breastfeed. Intravenous fluids are used for hydration, but total parenteral nutrition (TPN) is not readily available. Past work suggests that 4.2% of deliveries at KCMC result in a perinatal loss, with stillbirths representing nearly twice as many losses as neonatal deaths [30]. This degree of perinatal loss is equivalent to a rate of 41.6 deaths per 1000 total births at KCMC, reflecting its position as a referral center seeing a high volume of complex cases. This level of perinatal loss is higher than the composite stillbirth rate of 35.1 and neonatal mortality rate of 20.1 reported for the sub-Saharan Africa region [31], and is well above the target of 12.0 for both perinatal loss indices as set by the WHO [5].

### Research participants

A convenience sample of nurses, midwives, junior and senior physicians were invited to complete the KAP survey over a 4-month period in 2022. Participants were selected based on their availability during the study period and their willingness to complete the survey. Inclusion criteria were the ability to read and write in either English or Kiswahili and at least 3 months of experience working in their respective unit caring for pregnant women or newborn infants. The 3-month requirement was felt to represent the minimum amount of time necessary to contextualize one’s views on patient care and family support within operating procedures of a given hospital unit. A goal of 60 survey participants was selected a priori based on an assessment of feasibility given the number of staff employed at KCMC while allowing for a diversity of views to be obtained from members of various clinical roles.

At inception, the survey was administered in the Pediatric, Obstetrics and Gynecology, and Emergency Medicine (EM) Departments. However, early feedback from EM providers approached for the study revealed that most do not have sufficient exposure to labor and delivery or immediate newborn care to accurately complete the survey. Five EM providers with significant exposure to obstetric and pediatric care were included, but data collection in the EM department was discontinued early due to low numbers of healthcare providers with sufficient relevant clinical exposure.

### Survey development

KAP survey questions were co-developed by the US and Tanzanian members of the research team. Content was drawn from an extensive literature review on perinatal loss in LMICs and from local expert opinion. The structure of the KAP survey was based on similar survey work performed at KCMC [32]. The KAP survey was independently translated from English to Kiswahili by two native speakers. Translations were then checked for accuracy and retention of meaning by a team of Kiswahili speakers. Additional survey refinement occurred in order to arrive at the intended meaning, within local context, of each question. Prior to use, the completed survey was pilot tested by three non-clinical members and two clinical members of the research team.

### Data collection

Written consent was obtained prior to administering each survey and participants were offered a written copy of the consent and study description. Surveys were administered on an electronic tablet directly into REDCap (Research Electronic Data Capture) or on paper printouts and transposed into REDCap by a study team member. All study data were managed in the REDCap electronic data capture tool hosted at Duke University [33,34]. REDCap is a secure, web-based software platform designed to support data capture for research studies, providing 1) an intuitive interface for validated data capture; 2) audit trails for tracking data manipulation and export procedures; 3) automated export procedures for seamless data downloads to common statistical packages; and 4) procedures for data integration and interoperability with external sources. Surveys displayed questions in both English and Kiswahili.

Recruitment and data collection took place from 8 Aug 2022 through 11 Nov 2022. Each participant completed a written consent process detailing the purpose and intended use of data obtained from this study. All participants were above the age of 18.

### Analysis

Data was exported from REDCap for quantitative analyses using the R Software for Statistical Computing (version 4.1.0) [35] in R studio (version 2022.12.0) [36]. The dplyr and tidyr packages were used within R studio. We used simple descriptive statistics to describe responses to KAP surveys. A series of two-sample z-tests were conducted to determine if there were statistically significant differences in agreement rates for posed stillbirth definitions between obstetric and pediatric providers, between physicians and non-physician respondents, between providers with less or greater than 5 years of clinical experience, and for male vs female providers. Analysis was limited to stillbirth definitions that received at least 15 total responses for a given comparison sample. A significance level (α) of 0.05 was used in this analysis.

### Ethics

Ethics approval for this study was obtained from Duke University (Pro00112549-INIT-1.0), Kilimanjaro Christian Medical Center/ Kilimanjaro Christian Medical University College (certificate #1357), and the National Institute for Medical Research in Tanzania (NIMR/HQ/R.8a/Vol.IX/4029). All data are shared within the body of this publication. To ensure confidentiality for respondents, all collected data is stored on a secure server and used for its stated intended purpose only. Consent was obtained before data collection, as described above.

## Results

A total of 74 healthcare providers completed the KAP survey. Of those, 41 were pediatric providers, 28 were obstetrics and gynecology providers, and 5 were emergency medicine providers. The majority of participants (64.9%) were physicians, with nurses (21.6%) and midwives (13.5%) comprising the remainder. The majority of participants (n = 44, 59.5%) were female providers. Nearly all respondents had been working in healthcare for 10 years or less (n = 70, 94.5%) (Table 1). All providers were asked the number of stillbirths and neonatal deaths they personally witnessed in the past 12 months. Obstetrical providers observed a similar number of stillbirths (mean 11.5, median 8.5) and neonatal deaths (mean 13.2, median 8.5). Pediatric providers observed a higher number of neonatal deaths (mean 32.4, median 20.0) compared to stillbirths (mean 5.0, median 1.0). Within obstetrics, midwives had the greatest exposure to both forms of perinatal loss as well as the widest range of responses. Responses across roles and specialties were skewed in distribution; medians and interquartile ranges are presented in Table 2.

**Table 1:** Demographics of survey respondents (N = 74).

Role	N (%)
Doctor	48 (64.9%)
Nurse	16 (21.6%)
Midwife	10 (13.5%)
**Department**	
Pediatrics	41 (55.4%)
Obstetrics	28 (37.8%)
Emergency	5 (6.8%)
**Time working in Healthcare**	
< 5 years	48 (64.9%)
5–10 years	22 (29.7%)
11–20 years	2 (2.7%)
> 20 years	2 (2.7%)
**Gender**	
Male	30 (40.5%)
Female	44 (59.5%)
Other	0
**Age (Years)**	Mean: 31.7 Standard Deviation: 9.2 Range: 22–78

**Table 2:** Provider exposure to perinatal loss (N = 67).

Personal Exposure to Stillbirth and Neonatal Death					
	Obstetrics			Pediatrics	
	Doctor (n = 19)	Nurse (n = 2)	Midwife (n = 7)	Doctor (n = 28)	Nurse (n = 11)
**Stillborn infants seen in the past 12 months** Median (IQR)	6.0 (5.0)	6.5 (3.5)	10.0 (23.5)	2.0 (6.3)	0.0 (4.5)
	8.5 (5.5)			1.0 (5.5)	
**Neonatal deaths seen in the past 12 months** Median (IQR)	6.0 (14.0)	6.0 (4.0)	10.0 (24.0)	23.0 (36.3)	15.0 (43.0)
	8.5 (17)			20.0 (42.5)	

*IQR: Interquartile Range.

Providers were then presented with a series of clinical descriptions and asked which of these descriptions would be classified as a stillbirth at KCMC. Respondents could select any and all statements with which they agreed, including not selecting any statements. The most commonly selected clinical description, chosen by 67.6% of clinicians, was “An infant born without any signs of life at the time of delivery.” The second most commonly selected description was “An infant born who had a detectable heartbeat just prior to delivery but no detectable heartbeat after delivery.” This description was chosen by 25.7% of respondents (Table 3). These two stillbirth classification descriptions had 15 or more affirmative responses across selected comparator groups. A series of two-sample z-tests evaluating agreement rates for these definitions aligning with appropriate classification of a stillbirth did not demonstrate any significant differences between evaluated groups. Compared groups included 1) obstetric and pediatric providers (p = 0.385 for description 1 and p = 0.461 for description 2), 2) doctors and nurses / midwives (p = 0.463 and p = 0.712), 3) clinical experience < 5 years and clinical experience ≥ 5 years (p = 0.828 and p = 0.206), and 4) male gender and female gender (p = 0.513 and p = 0.715). Responses for the remaining four potential stillbirth descriptions ranged from 25.7% agreement to 8.1% agreement. Statistical comparisons of response patterns were not performed for these stillbirth definitions options given the low response rate for descriptions 3–6.

**Table 3:** Provider agreement with stillbirth classification descriptions.

Stillbirth Classification Description	All (n = 74)		Clinical Specialty				Clinical Role				Clinical Experience				Gender			
			Obstetrics (n = 28)		Pediatrics (n = 41)		Doctor (n = 48)		Nurse / Midwife (n = 26)		<5 Years (n = 48)		≥ 5 Years (n = 26)		Male (n = 30)		Female (n = 44)	
	n	%	n	%	n	%	n	%	n	%	n	%	n	%	n	%	n	%
1) An infant born without any signs of life at the time of delivery	50	67.6	20	71.4	25	61.0	31	64.6	19	73.1	32	66.7	18	69.2	19	63.3	31	70.5
2) An infant born who had a detectable heartbeat just prior to delivery but no detectable heartbeat after delivery	19	25.7	6	21.4	12	29.3	13	27.1	6	23.1	10	20.8	9	34.6	7	23.3	12	27.3
3) Death within minutes after delivery for a preterm infant that was not expected to survive	14	18.9	3	10.7	10	24.4	7	14.6	7	26.9	11	22.9	3	11.5	7	23.3	7	15.9
4) Death of a fetus < 500g within an hour of birth	9	12.2	2	7.1	6	14.6	6	12.5	3	11.5	7	14.6	2	7.7	3	10.0	6	13.6
5) Death of an infant who was born with a detectable heartbeat but does not survive resuscitation in the delivery room	7	9.5	1	3.6	5	12.2	4	8.3	3	11.5	3	6.3	4	15.4	1	3.3	6	13.6
6) Death after delivery of an infant delivered in the setting of an induced abortion	6	8.1	1	3.6	5	12.2	4	8.3	2	7.7	5	10.4	1	3.8	1	3.3	5	11.4

**Fig 1:**
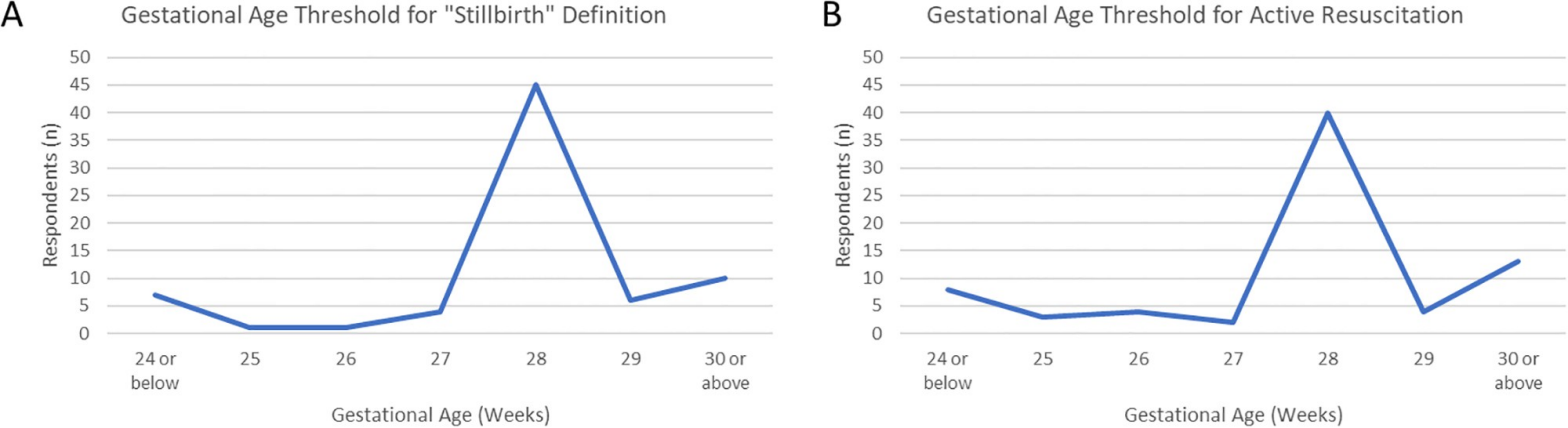
Gestational age thresholds selected by providers. Selected thresholds to A) define a stillbirth (vs an “abortus”) and B) provide active resuscitation via CPAP, PPV, or intubation.

Providers were next asked the minimum gestational age for defining a stillbirth. Most, 45 providers (60.8%), identified 28 weeks as the threshold. Sixteen providers selected the higher thresholds of 29 or 30 weeks, while 13 selected gestational ages between 24 and 27 weeks (Fig 1A).

When asked about thresholds for intervention, 40 providers (54.1%) said the youngest gestational age at which they would provide “active resuscitation”–defined as the provision of continuous positive airway pressure (CPAP), positive pressure ventilation (PPV), or intubation–was at 28 weeks gestation. A minority, 13 providers (17.6%), would withhold such resuscitation until 30 weeks or greater, while 8 providers (10.8%) would offer resuscitation at 24 weeks and younger (Fig 1B). In justifying their selection, a majority of providers (41 respondents, 55.4%) selected “there is a low chance of survival below this point”. Twenty providers cited national guidelines and 13 cited WHO recommendations as additional support. No provider selected the option “the family would not want me to resuscitate below this point” as a potential reason for using the 28-week threshold. When asked to consider decision making during resuscitations, 63.5% of providers stated that physicians are the sole decision makers about resuscitation. One third (33.8%) felt that doctors and parents engaged in shared decision making, and only 2 providers felt that parents were the primary decision makers for their newborn.

**Fig 2:**
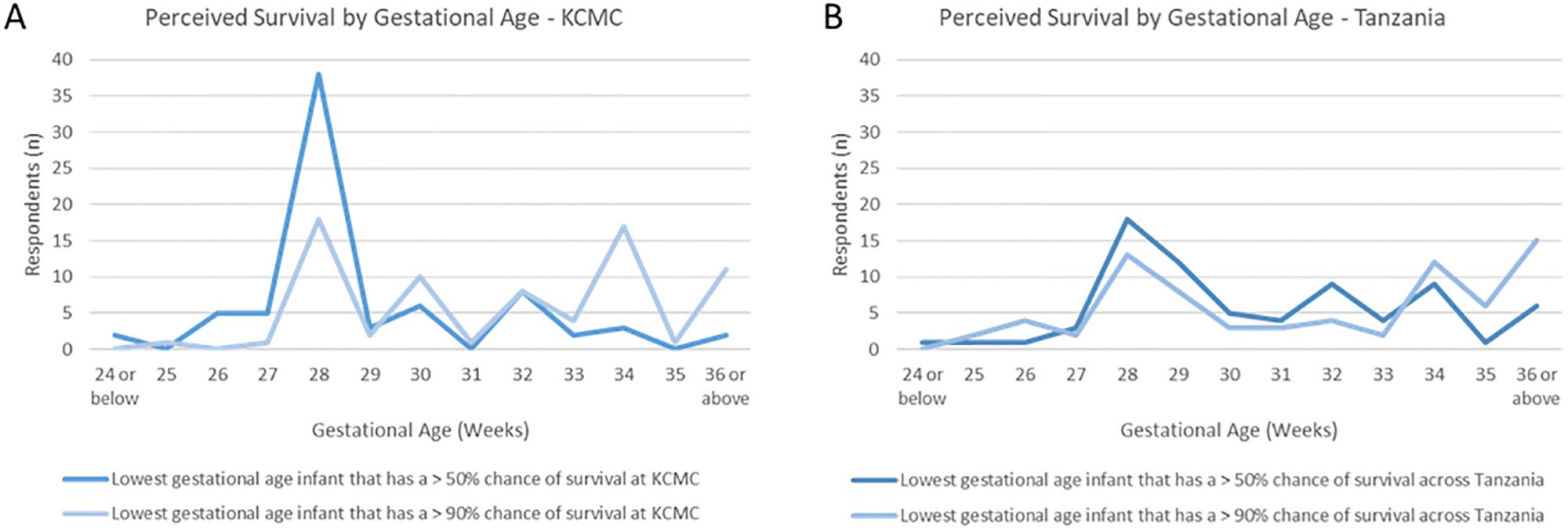
Perceived likelihood of survival by gestational age. Selected thresholds to A) predict 50% or 90% survival at KCMC, and B) predict 50% or 90% survival across Tanzania.

Next, providers were asked about their expectations for neonatal survival both at KCMC and “across Tanzania”. Obstetric and pediatric clinicians indicated the gestational age, in weeks, where they thought an infant had a 50% chance of survival and a 90% chance of survival in each of these settings. At KCMC, 38 healthcare providers (51.4%) felt that a 50% survival occurred at 28 weeks. For 90% survival at KCMC, 28 weeks remained the dominant response with 18 providers (24.3%) selecting this gestational age; however, 34 weeks was a close second choice and was selected by 17 providers (23%). In regard to anticipated survival across Tanzania, 18 providers (24.3%) felt that a 50% survival occurred at 28 weeks, with a majority selecting gestational ages 29 weeks or greater. For 90% survival, “36 weeks or later” was the most common selection (n = 15, 20.3%), followed by 28 weeks (n = 13, 17.6%). (Fig 2A and 2B).

**Fig 3:**
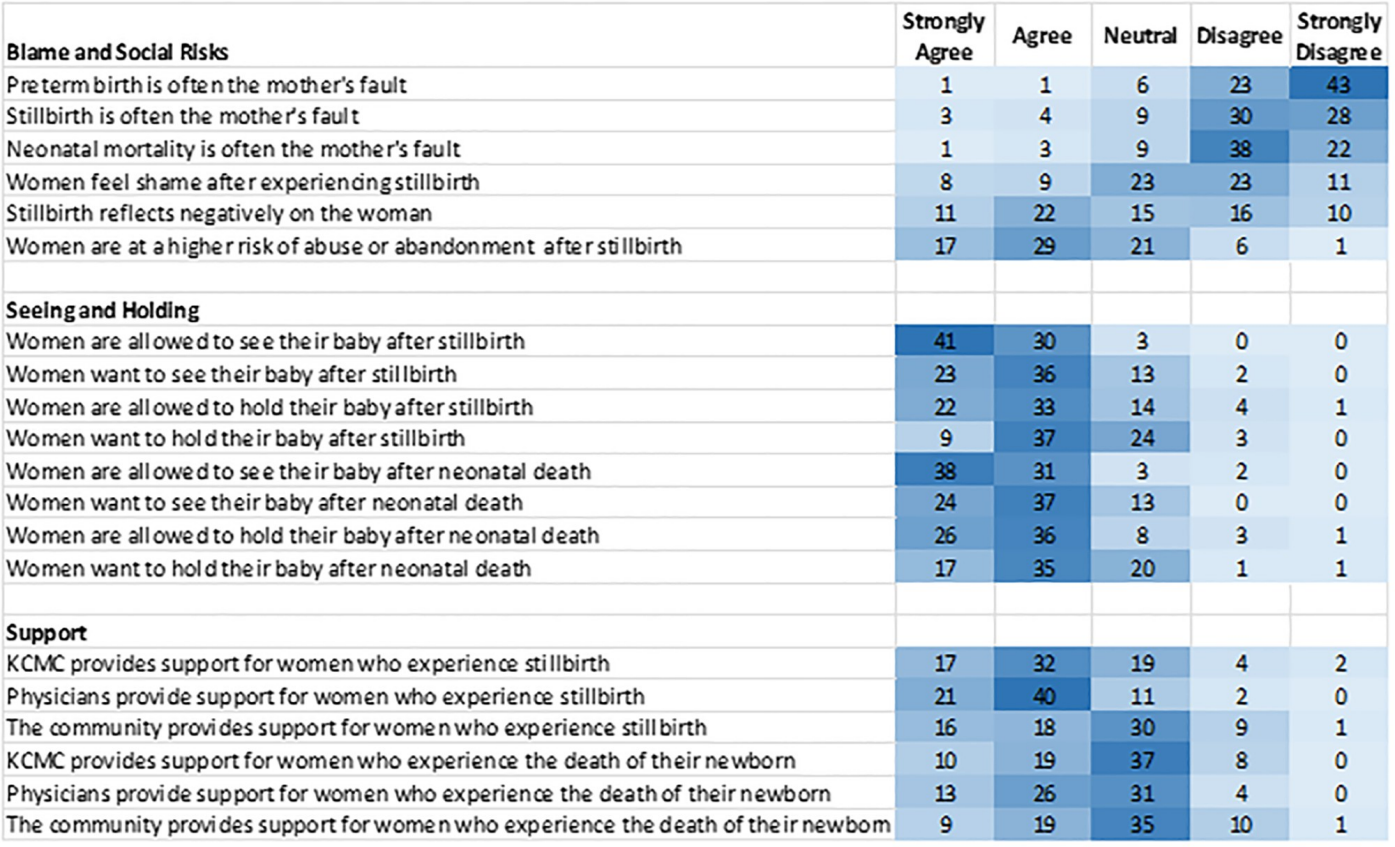
Level of agreement with statements addressing practices around perinatal loss. The number of providers with each response is displayed, with density shading indicating the most common responses (dark color).

The KAP survey also contained questions addressing provider beliefs about stillbirth and neonatal death. The majority (n = 43, 58.1%) of respondents felt that stillbirth and neonatal death were equally poor outcomes. The remainder of providers were nearly equally split (n = 16 vs 15) on if stillbirth or neonatal death was the worse outcome. The majority of providers disagreed or strongly disagreed that preterm birth, stillbirth, and neonatal mortality were the mother’s fault (89.2%, 78.4%, and 81.1%, respectively). However, nearly half (44.6%) felt that stillbirth “reflects negatively on the woman”. An even greater number (62.2%) agreed or strongly agreed that women are “at a higher risk of abuse or abandonment” after stillbirth (Fig 3). Overall, there was a positive attitude towards a woman holding her infant following stillbirth or neonatal death. Following stillbirth 95.9% of providers said women are allowed to see their baby and 74.3% said women are allowed to hold their baby. Within each category fewer providers, 79.7% and 63.0%, respectively, agreed or strongly agreed that women wanted to see or hold their baby. For neonatal death, 93.2% of providers said women were allowed to see their baby and 83.8% said women were allowed to hold their baby. As with stillbirth, agreement rates dropped to 82.4% and 70.3% when asked if women wanted to see and hold their baby after death. Lastly, more providers (66.2%) felt that “KCMC provides support for women” who experience stillbirth as composed to those who experience neonatal death (39.2%). A minority of respondents felt that the local community provides support for women after stillbirth (45.9%) or neonatal death (37.8%).

## Discussion

Despite the substantial global burden of neonatal mortality and stillbirth in LMICs, little is known about how healthcare providers view and approach stillbirth and neonatal death. Here, we explore perinatal loss related knowledge, attitudes, and practices of obstetric and pediatric providers at a tertiary care delivery center in Moshi, Tanzania. How providers describe perinatal loss and conceptualize the maternal experience of stillbirth or neonatal death helps us understand the stance from which healthcare workers approach perinatal loss. This perspective is important for those hoping to create both provider and parent-focused interventions. Our KAP survey findings can be used to inform future intervention development by highlighting the need for more standardized classification of perinatal loss, reflection on gestational age outcomes, and provider skill development focused on supporting the psychosocial needs of women who suffer perinatal loss.

All healthcare providers in our survey cohort reported clinical experience with both stillbirth and neonatal death. Not surprisingly, obstetric providers witnessed a greater number of stillbirths compared to their pediatric colleagues. Likewise, pediatric physicians and nurses had more experience with neonatal death than did the obstetrical team members. Interestingly, however, both physicians and nurses on the obstetrical service reported similar rates of exposure to neonatal death as to stillbirth. This may be due to true higher rates of neonatal death compared to stillbirth reported at KCMC, or due to an underreporting or misclassification of stillbirths. Regardless, the shear burden of exposure to perinatal loss reported by our survey participants is impressive; the average provider in our survey would interface with over a dozen mothers a year who experienced either stillbirth or neonatal death. In order to best meet the physical and socio-emotional needs of these women, providers must have the empathic communication skills to explain the loss to mothers and offer follow-up support. In parallel, providers may need an avenue for debriefing and handling the potential emotional and mental burden of confronting such much loss in the work-place [37].

A basic component of improving perinatal care, communication, and outcome tracking is appropriately classifying perinatal losses as neonatal deaths or stillbirths. Variation in classifying these losses exists amongst national and international reporting systems, including the comprehensiveness of the systems and their reliability [14]. This may translate into confusion at the provider-level. Indeed, when our survey respondents were asked to indicate agreement with posed definitions of stillbirths, there was wide variation in classifications used. A significant proportion of survey respondents from KCMC did not affirm widely accepted definitions of stillbirth (“An infant born without any signs of life at the time of delivery” and “An infant born who had a detectable heartbeat just prior to delivery but no detectable heartbeat after delivery”). Similarly, many providers indicated definitions that would be classified as “live births”, rather than stillbirths, by leading health bodies such as the WHO [38], the Centers for Disease Control (CDC) [39], and the American College of Obstetrics and Gynecology (ACOG) [40]. These findings were true for both pediatric and obstetric providers and held true for both physician and non-physician respondents. Within our study, the lack of a shared understanding of how stillbirths are defined institutionally and nationally raises concerns about the appropriate classification of stillbirths within vital records databases and the framing of perinatal loss used by providers when engaging with bereaved families. This challenge is not unique to our study population. A 2021 report from the WHO elevated this concern, noting that most stillbirths and half of all neonatal deaths globally do not receive a birth certificate and are not registered in any viral records system [41]. The WHO further noted that recognizing and recording these perinatal losses is a key component in understanding the underlying contributors to perinatal loss.

Central to outcome reporting is an understanding of the role gestational age plays in both data tracking and clinical decision making. For decades, the WHO has used a 28-week threshold for international reporting of stillbirth and neonatal mortality [15]. While a standardized threshold allows for domestic and cross-national comparison of perinatal outcomes, this threshold was not meant to guide clinical practice around the provision of care. Several questions in our KAP survey addressed gestational age thresholds. Specifically, we asked about the threshold for defining a stillbirth, for offering active resuscitation, and for survival likelihoods of 50% and 90% at KCMC and across Tanzania. Across all of these questions, 28 weeks’ gestation bore out as a dominant response. With an increasing number of infants surviving below 28-weeks, few countries are currently applying the 28-week threshold in practice [42]. Even many LMICs report admitting gestationally younger infants to their NICUs and providing them with life-saving interventions [16,43,44]. Globally, data tracking for stillbirth and neonatal mortality rates now includes data on “extremely preterm infants”, defined as infants born between 22 and 28 weeks gestation [45]. These infants represent 4% of global preterm births[46] and an important subgroup of NICU patients in both high income countries (HICs) and LMIC settings. Importantly, preterm infants born before 28 weeks are unlikely to survive without active resuscitation, including respiratory support. Prior work from the United States has demonstrated that survival at a given gestational age is directly related to the provision of active resuscitation [47]. With the majority of providers in our survey cohort stating that they would not provide CPAP, PPV, or intubation to infants younger than 28 weeks, they essentially manifest a self-fulfilling prophecy wherein these children do not survive. It is therefore not surprising that the perceived likelihood of survival before 28 weeks is low, regardless of clinical context. This perception also aligns with global data on survival likelihoods; In HICs there is a 50% chance of survival at 24 weeks gestation, whereas the same odds do not occur until 34 weeks in many LMICs [15].

Globally, the literature on perinatal loss has emphasized the need for better bereavement support in LMICs [48–50]. Parent-focused studies in HICs highlight the importance of healthcare providers recognizing the deceased newborn as member of the family and engaging in “memory making” [26,51,52]. This process, which ranges from seeing and holding the infant after death to creating hand-molds and photographs of the infant, is less common in SSA. Earlier literature from the region calls out the societal, and sometimes personal, desire not to retain memories of a neonatal death. For example, in Ethiopia, women are traditionally not permitted to hold their baby after suffering a perinatal loss in order shield the woman from emotional and mental pain [53]. Similarly, in Ghana, avoiding thinking about neonatal losses is thought to protect women from psychological harm [54]. Indeed, by emotionally and physically separating the mother from the newborn, some healthcare providers in SSA believe they are protecting and helping the woman [53,54]. However, these beliefs and practices may not align with what women want, especially in more recent years. One study from Nigeria showed that only half of women experiencing stillbirth were allowed to see the body of their infant and none were given the opportunity to hold the infant, although many would have liked to do so [55]. Our finding that most providers believe women want to see and hold their infant following stillbirth or neonatal death echoes this newer view of post-loss engagement with the infant’s body. This important topic warrants further study that incorporates the viewpoints of parents to assess if their desires align with the perceived desires noted by providers in our study.

Lastly, our study captured the important topic of the stigma associated with perinatal loss. Unfortunately, in many settings, the suffering experienced by women after a perinatal loss may be compounded by being blamed for that loss. Work from across SSA reveals a prevailing belief that the mother is somehow to blame for the premature death of her child [53,56,57]. While in our study, approximately 80% of providers disagreed with this assignment of blame, the remaining 20% were neutral or in agreement with the mother bearing responsibility for her perinatal loss. Moreover, nearly half of those surveyed agreed that stillbirth reflected negatively on the woman. This belief stands in contrast to a registry based studies at KCMC showing that preeclampsia and placental abruption are the maternal conditions most strongly associated with stillbirth [29] and birth asphyxia and preterm delivery are the leading causes of neonatal death [58]. Blame towards the mother in these situations is inappropriate. Indeed, within Tanzania, research shows that poor communication and lack of empathy from health workers contributes to impaired grieving following stillbirth [59]. Similarly, nearly two-thirds of our surveyed providers believed that women were at risk of social or physical harm following stillbirth; this further underscores the pervasiveness of perinatal-loss stigma. This stigma leads to the normalization or societal expectation that women experience additional consequences at home or in their community following their loss. For this reason, there has been increased recognition of the importance of psychosocial support for families following perinatal loss [23,49,60]. This recognition comes alongside global calls to sensitize healthcare workers to parents who have had a stillbirth concurrent with efforts to increase the number of births attended by a skilled birth attendant [60].

Methodological limitations of this study include the short timeframe for data collection and the inclusion of just one clinical setting in SSA. This limits the potential applicability of findings to other contexts, including lower-level facilities in Tanzania and care settings across SSA. Secondly, we did not ask providers about past exposure to perinatal loss prior to enrolling them in the study. This raises the possibility that providers did not have sufficient exposure to answer the questions; however, all providers did have a minimum of 3 months of experience working in their respective unit, thereby minimizing the likelihood of insufficient exposure to perinatal loss. Within the survey itself, there is the possibility that on “select all that apply” questions that providers mistakenly only selected one response, not realizing they could select several options. This concern is higher on the paper-administered surveys, but the risk was minimized by clear instructions in both Kiswahili and English. Next, we must recognize that this survey focused on the knowledge, attitudes, and practices of healthcare providers. The perceptions of medical professionals about the lived experience of women after perinatal loss may not mirror the essential viewpoints of these women. For example, while a large number of providers in our study called out the risk of abuse and abandonment in women who experience perinatal loss, we cannot extrapolate that to represent the actual experience of these women. Lastly, our study did not address the perceived experiences of fathers after perinatal loss nor how the healthcare system can support the broader family after stillbirth or neonatal death. Work that integrates the provider perspective with the lived experience and beliefs of parents is needed to address this gap.

## Conclusions

Ours is one of a handful of studies detailing the beliefs and practices of healthcare providers who treat and bear witness to perinatal loss in SSA. Our work suggests that a deeper understanding of how perinatal loss is approached in delivery settings and via social-support mechanisms is needed in order to best meet the needs of bereaved women. Our findings highlight the need for improved mental health resources for families following perinatal loss. Indeed, it is imperative that parallel global efforts be directed towards improving rates of perinatal loss and strengthening the capacity of health systems to care for and provide psychosocial support for parents. A core element of this systems strengthening involves working with healthcare providers to better classify perinatal loss and respond to the socio-emotional needs of parents. Further work should explore maternal and family viewpoints on perinatal loss and bereavement and work to inform thoughtful and culturally appropriate tools to better support families after stillbirth and neonatal death.

## Supporting information

S1 Checklist (DOCX)
